# Genetic consequences of long‐term small effective population size in the critically endangered pygmy hog

**DOI:** 10.1111/eva.13150

**Published:** 2020-11-11

**Authors:** Langqing Liu, Mirte Bosse, Hendrik‐Jan Megens, Manon de Visser, Martien A. M. Groenen, Ole Madsen

**Affiliations:** ^1^ Animal Breeding and Genomics Wageningen University & Research Wageningen the Netherlands

**Keywords:** conservation genomics, deleterious variants, inbreeding, population genomics

## Abstract

Increasing human disturbance and climate change have a major impact on habitat integrity and size, with far‐reaching consequences for wild fauna and flora. Specifically, population decline and habitat fragmentation result in small, isolated populations. To what extend different endangered species can cope with small population size is still largely unknown. Studies on the genomic landscape of these species can shed light on past demographic dynamics and current genetic load, thereby also providing guidance for conservation programs. The pygmy hog (*Porcula salvania*) is the smallest and rarest wild pig in the world, with current estimation of only a few hundred living in the wild. Here, we analyzed whole‐genome sequencing data of six pygmy hogs, three from the wild and three from a captive population, along with 30 pigs representing six other *Suidae*. First, we show that the pygmy hog had a very small population size with low genetic diversity over the course of the past ~1 million years. One indication of historical small effective population size is the absence of mitochondrial variation in the six sequenced individuals. Second, we evaluated the impact of historical demography. Runs of homozygosity (ROH) analysis suggests that the pygmy hog population has gone through past but not recent inbreeding. Also, the long‐term, extremely small population size may have led to the accumulation of harmful mutations suggesting that the accumulation of deleterious mutations is exceeding purifying selection in this species. Thus, care has to be taken in the conservation program to avoid or minimize the potential for further inbreeding depression, and guard against environmental changes in the future.

## 1 INTRODUCTION

During the last glacial maximum, the ranges of most temperate species shifted and shrunk as temperatures decreased (Davis & Shaw, 2001). During the Holocene, human populations expanded rapidly, and negatively affected biotic recoveries and natural range expansions through both hunting and land clearing (Ellis, 2015). Thus, the combined effects of climatic changes and human activities have reduced population sizes of many species throughout the world to a critically small size over the past 10,000 years (Miraldo et al., 2016; Pimm & Raven, 2017).

Small, fragmented, and isolated populations lead to reduced genetic variation and increased inbreeding and genetic drift (Lynch et al., 1995, 2016). Inbreeding can have a negative effect on population viability through inbreeding depression, which is a consequence of an increase of harmful mutations in the homozygous state in inbred individuals (Kardos et al., 2017; Pekkala et al., 2012, 2014). In some populations, purging of harmful mutations can result in lower load. Purifying selection facilitated by inbreeding as it increases the homozygosity of partially recessive deleterious variants (Hedrick & Garcia‐Dorado, 2016). However, in extremely small populations, genetic drift tends to prevail over natural selection, limiting the potential for purifying selection against deleterious variation, and even allowing deleterious variants to increase in frequency (W. C. Funk et al., 2016; Lynch et al., 2016). Importantly, low levels of genetic variation are expected to reduce the opportunities for selection and to limit adaptive potential in populations that experience rapid environmental changes, for example, new diseases and climate fluctuation (Hamilton & Miller, 2016; Piertney & Oliver, 2006).

Studies on demographic history and erosion of genomic variation of endangered populations can show the impact of losing genomic diversity and accumulation of genetic load. For instance, in the endangered Cheetah (*Acinonyx jubatus*) population, long‐term decline and subsequent bottlenecks have resulted in excessive deleterious mutations, reducing reproductive success (Dobrynin et al., 2015; Merola, 1994). However, not all populations with low genetic diversity suffer from inbreeding depression. Similar patterns of long‐term decline are apparent in the genomes of island foxes, which resulted in extensive runs of homozygosity and increased genetic load. Yet, the lack of apparent phenotypical defects suggests that deleterious variants were purged from the island fox population in parallel with further adaptation to the local environment (Robinson et al., 2016, 2018). It is, therefore, important to understand demographic history as well as temporal changes in mutational load in small, fragmented populations in order to predict the impact of inbreeding and increase the chances of long‐term population persistence.

The pygmy hog (*Porcula salvania*) is the smallest and the rarest wild suid in the world, and so far known as the sole living representative of the genus *Porcula*. The pygmy hog has been classified as a critically endangered species by the International Union of Conservation of Nature (IUCN) since 2008. The pygmy hog is confined to the tall grass savanna of the Himalayan foothills. Since the early 20th century, human settlement and agriculture led to accelerated fragmentation and loss of pygmy hog habitat (Peet et al., 1999). The pygmy hog was believed to be extinct in most of its natural range in the Terai and Duars region (Oliver & Deb Roy, 1993) until they were rediscovered in 1971. Currently, only one viable wild population remains, in Manas National Park, northern Assam, India. Considering its critical status and the unique habitat it lives in, a recovery program for this species, the Pygmy Hog Conservation Programme (PHCP), was initiated in 1995 (PHCP, 2008). Starting with six wild‐caught hogs, the breeding program exceeded early expectations. The captive population is now around 80 (Huffman, 2016). Although the PHCP has benefited from several decades of planned breeding and pedigree management, so far there has been no information on the genetic diversity in the individuals that were used to establish the breeding program. This information is essential to inform the breeding program to prevent inbreeding issues.

It is still largely unknown whether the small population has experienced purifying selection of harmful mutations and whether current inbreeding leads to inbreeding depression in this population. To infer their demographic history, and eventual inbreeding concerns, we studied whole‐genome data of six pygmy hogs: three from the wild and three from the breeding program. By comparing the pygmy hog information with 30 pigs belonging to six other old‐world pig species (Table S1), we interpreted our findings in the context of these other pig species, whose demographic history has been well studied. For example, we included the critically endangered Javan warty pig (*Sus verrucosus*), which is highly inbred due to recent zoo management (Semiadi & Meijaard, 2006). We also include much more widespread species, such as the European wild boar (*Sus scrofa*), which have experienced profound population bottlenecks due to glaciations and, historically, hunting and habitat loss (Groenen et al., 2012).

In this study, we aim at using a comparative genomics approach to infer past population dynamics and assess the consequences of severe population decline. Our results provide a detailed genomic estimation of the pygmy hog's population history, genomic diversity, inbreeding status, and genetic load. These results provide a strong foundation in evaluating the conservation status of the pygmy hog and highlighting the importance of genomic monitoring in population management of pygmy hogs and other endangered species, both in situ and ex situ.

## 2 MATERIALS AND METHODS

### 2.1 Whole‐genome resequencing, variant calling, and filtering

The pygmy hog samples used for this research are derived from three wild and three captive individuals. On these samples, whole‐genome Illumina PE 100 bp resequencing was performed at SciGenom Laboratories in Chennai, India. A selection of other *Suidae* species was included (Table S1). All these samples were also sequenced with the Illumina sequence technology. The whole‐genome sequencing data were trimmed using sickle (Version 1.33, https://github.com/najoshi/sickle) with default parameters. The trimmed reads were aligned to the Sscrofa 11.1 reference genome. Since there are multiple closely related species to the reference species, we used the unique alignment option of MOSAIK aligner (Version 2.2.30) (Lee et al., 2014) to increase mapping accuracy (Pightling et al., 2014). Local re‐alignment was performed using GATK (Version 3.7) RealignmentTargetCreator and IndelRealigner and variants were called using GATK UnifiedGenotyper (McKenna et al., 2010), with the –stand_call_conf option set to 50, the –stand_emit_conf option set to 20, and the ‐dcov option set to 200. Variants with a read depth between 0.5 and 2.0 times of the average sample genome coverage were selected and stored in variant calling format (Table S1).

### 2.2 Mitochondrial genome assembly and analysis

As no pygmy hog mitochondrial sequence was available, we reconstructed one, using the short‐read data from the high‐coverage individual (Table S1). We assembled the mitochondrial genome through iterative mapping using MITObim v1.8 (Hahn et al., 2013) on 100 million trimmed and merged reads, subsampled using seqtk (version 1.3 r106), https://github.com/lh3/seqtk. Mitochondrial reconstruction was performed in three independent runs using three different starting bait reference sequences. The references included the domestic pig (AF034253.1), common warthog (DQ409327.1), and cattle (AY526085.1). We implemented MITObim using default parameters apart from mismatch value where we used zero. We resolved the circularity of mitochondrial DNA using the published control region sequences (S. M. Funk et al., 2007). All three independent MITObim assembly runs produced identical pygmy hog mitochondrial sequences, providing strong evidence that our reconstructed mitochondrial genome is correct. The reconstructed mitochondrial genome served as a reference sequence for subsequent mitochondrial DNA mapping analyses. We mapped the trimmed and merged reads from our 6 pygmy hogs to the reconstructed reference sequence using BWA‐mem (version 0.7.15) (Li & Durbin, 2009) using default parameters and parsed the mapped files using SAMtools (version 0.1.19‐44428cd) (Li et al., 2009). Local re‐alignment was performed using GATK RealignmentTargetCreator and IndelRealigner and variants were called using GATK UnifiedGenotyper (McKenna et al., 2010), with the –stand_call_conf option set to 50 and the –stand_emit_conf option set to 20. The consensus sequences were constructed using ANGSD (version 0.929) (Korneliussen et al., 2014). Mitochondrial genome sequence was aligned and analyzed using MEGA7 (Kumar et al., 2009).

### 2.3 Genetic diversity

Nucleotide diversity was calculated for bins of 10 kbp over the entire genome within each individual, following the description in Bosse et al. (2012). Nucleotide diversity was represented by “SNPbin”. “SNPbin” is the SNP count per 10 kbp window, corrected for the number of bases within that bin that was not covered after the read‐depth filtering, so that the eventual SNP count per bin (SNPbin) is proportional to 10,000 covered bases. SNP count is the total number of SNPs counted in a bin of 10 kbp. We assessed genetic diversity by calculating heterozygosity for each SNPbin, here defined as the number of heterozygous genotypes divided by the number of called sites within a single individual. Heterozygosity was calculated for the entire autosomal genome and in 100 kb sliding windows with a 10 kb step size. Windows with more than 20% of the sites failing the quality filters, or with fewer than 20 kb of confidently called sequence were excluded. Peaks of heterozygosity within a genome were defined as windows with heterozygosity greater than two standard deviations above the mean, based on the genome‐wide distribution of per‐window heterozygosity. Overlapping windows of high heterozygosity were merged using BEDTools (version 2.28.0) (Quinlan, 2014).

### 2.4 Runs of homozygosity (ROH) analysis

For homozygosity analysis, we calculated the runs of homozygosity (ROH) to estimate autozygosity for the sequenced individual. ROH for an individual were calculated based on the following criteria specified in Bosse et al. (2012) and using the python script specified in Bortoluzzi et al. (2019). This included the number of SNPs, in a window size of 10 Kb, counted below 0.25 times the average whole‐genome SNP count; and the homozygous stretches contained at least 10 consecutive windows which showed a total SNP average lower than the genomic average. Sufficiently covered windows with 0.5–2 times the individual average depth was considered. The relaxed threshold for individual windows was used within a homozygous stretch to avoid local assembly or alignment errors, which was done by allowing for maximum twice the genomic average SNP count, and the average SNP count within the candidate ROH to not exceed 1/4 the genomic average. The inbreeding coefficient derived from ROH genomic coverage (F_ROH_) was calculated by dividing total ROH length per individual by total genome length across all autosomes (~2.4 Gb) for each individual.

### 2.5 Variant annotation

All variants were annotated using Variant Effect Predictor (ensembl‐vep version 91.1) (Ihaka and Gentleman, 2015), with “‐‐species sus_scrofa ‐‐fork 4 ‐‐canonical ‐‐stats_text ‐‐gene_phenotype ‐‐numbers ‐‐domains ‐‐symbol ‐‐buffer_size 100000 ‐‐offline ‐‐force_overwrite ‐‐vcf ‐‐sift b”. Functional significance of amino acid substitutions was predicted using SIFT (Kumar et al., 2009). Putative deleterious mutation was further evaluated by pig Combined Annotation Dependent Depletion (pCADD) (Groß et al., 2020).

### 2.6 Functional, pathway and interaction enrichment analysis

ClusterProfiler (version 3.6.0) (Yu et al., 2012) was applied to perform GO analysis (including cellular composition, molecular function, and biological process terms) and Kyoto Encyclopedia of Genes and Genomes (KEGG) pathway enrichment analysis. False discovery rate (FDR) was performed to adjust *p*‐values using the Benjamini and Hochberg method. A *p* < .05 was used as the cutoff criterion.

### 2.7 PSMC analysis

To derive an estimate of the historical effective population size, a Pairwise Sequential Markovian Coalescence (PSMC version 0.6.4‐r49) model was used (Li & Durbin, 2011). This software uses the time to most recent common ancestor of a diploid genome (determined by looking at the density of heterozygotes) to estimate the effective population size (Ne) in the past. The individual whole‐genome consensus sequence, called by SAMtools (Li et al., 2009), was used as an input for this analysis. We used a generation time of 5 years (in concordance with the studbook files that showed a generation time for the captive population of 4.5 years) and a default mutation rate/generation of 2.5 × 10^–8^. The following parameters were used: T_max_ = 20; *n* = 64 (“4 + 50 × 1 + 4 + 6”).

### 2.8 Forward simulations of genetic variation in pygmy hog population

To evaluate the demographic parameters that could lead to purging of harmful mutations, we performed forward‐in‐time simulations as described in Robinson et al. (2018). We simulated neutral and deleterious variation under a constant population size, involving the establishment of a small population (*N* = 100, 200, 300, 500 or 1,000 individuals) derived from a large ancestral population (*N* = 10,000 individuals). Based on the PSMC results, the pygmy hog kept a low and stable population size from 100 ~ 200 kya. We assumed the generation time to be 5 years. The small populations were randomly sampled from the ancestral population and kept at constant size for 20,000 or 40,000 generations. Each simulated individual consisted of a diploid 2 Mb genome, consisting of 2,000 “genes” carried on 18 chromosomes proportional to chromosome lengths in the pig genome. A mutation rate of 2.5 × 10^–8^ and recombination rate were set to 0.8 cM/Mb (Tortereau et al., 2012). Each model was run for 20,000 and 40,000 generations following a 100,000 generation burn‐in period. 100 replicates were performed for each dominance value and each population size. The average number of alleles and the average number of homozygous alleles carried by each individual were calculated for deleterious (s < 0) and neutral mutations (s = 0). Deleterious mutations were grouped as strongly (s < −0.01), moderately (−0.01 < s < −0.001), and weakly deleterious (−0.001 < s < 0). One‐way ANOVA and Tukey HSD post hoc tests were used to evaluate significant differences in the number of total alleles and the number of homozygous alleles between different models.

## 3 RESULT

### 3.1 Relatedness between pygmy hog samples

**Figure 1:**
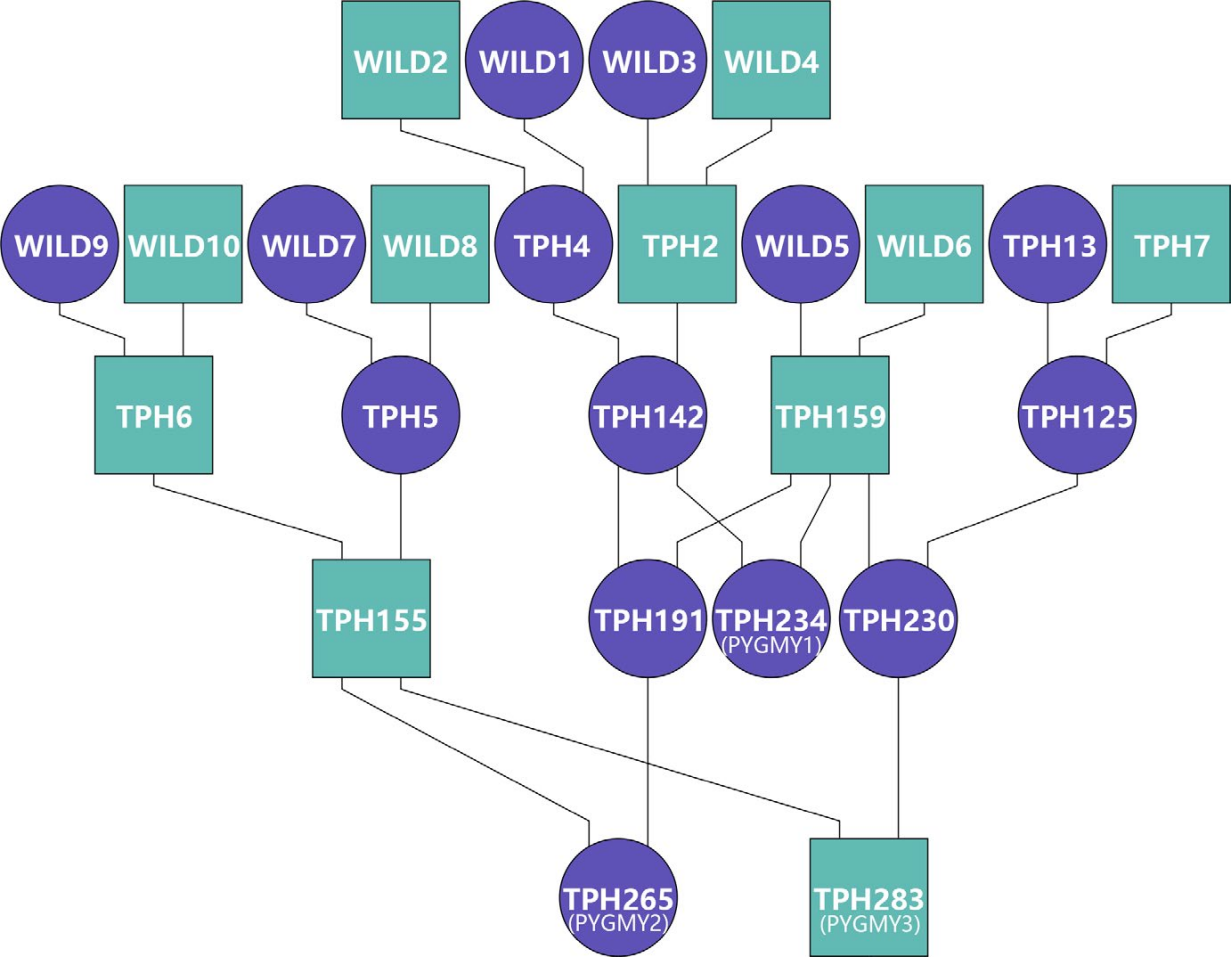
Studbook information. Part of the pedigree of the captive pygmy hog populations in the wild and the captive program reconstructed from the studbook files. Founder individuals were indicated as “WILD.” PYGMY1 (born 2007, sampled 2014); PYGMY2 (born 2008, sampled 2014); PYGMY3 (born 2009, sampled 2014)

According to pedigree information provided by the breeding program (Figure 1, Table S2), the three captive individuals were representatives of the third and fourth generations of the captive population. Assuming that all wild founders are not from the same family, no related mating caused by the breeding scheme was observed within the breeding program. However, two of the captive individuals (PYGMY2 and PYGMY3) are maternal half‐sibs, and with few founders inbreeding in future generations can be expected. Among all six pygmy hog samples, only a fraction, around 10%, of these variants was specific to either wild or captive individuals, and no significant difference between the heterozygosity levels between wild and captive animals was observed.

Due to maternal inheritance and lack of recombination, variation in mitochondrial genomes can provide unique insight into population structure. We assembled the complete mitochondrial genome from the wild‐caught individual with the highest read depth (Table S1). Next, we mapped the reads from the six individuals to the assembled mitochondrial sequence to assess the mitochondrial variation in our sequenced pygmy hogs. We observed that all six pygmy hogs carried identical mitochondrial genomes.

### 3.2 Genome‐wide diversity, inbreeding, and demographic history

**Figure 2:**
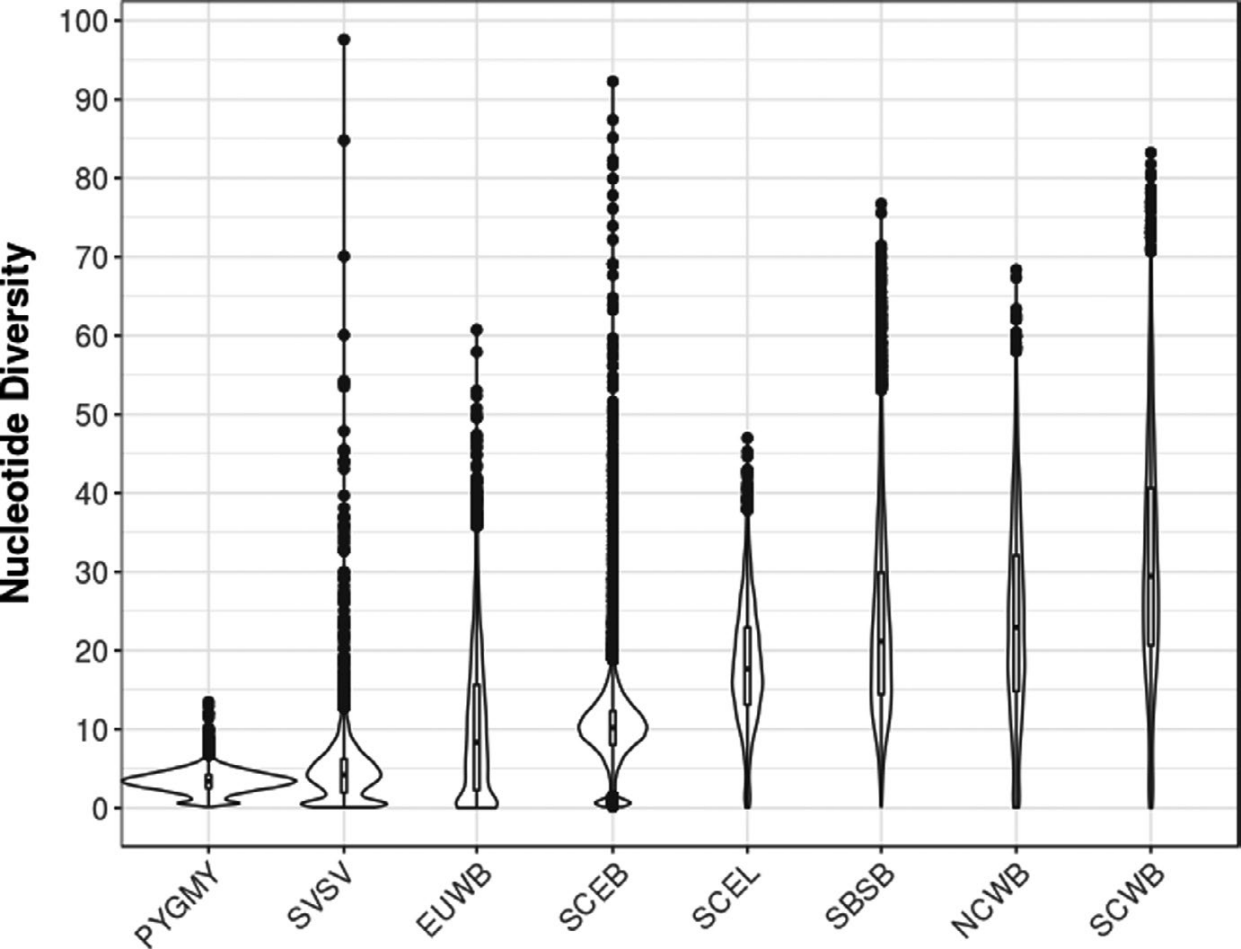
Nucleotide diversity (*10^−4 ^bp) in the sampled populations. PYGMY, pygmy hog; SBSB, Sus barbatus; SCEB, Sus cebrifons; SCEL, Sus celebensis; SVSV, Sus verrucosus; EUWB, European wild boar; NCWB, Northern China wild boar; SCWB, Southern China wild boar

We compared the genome‐wide autosomal nucleotide diversity between the pygmy hog and the other *Sus* species. Overall, genome‐wide nucleotide diversity (π) of the pygmy hog is much lower (3.33 ± 1.36) than for all the other *Suidae* species, which are less threatened (15.15 ± 10.30). This number is even lower than what is observed in *Sus verrucosus* (4.82 ± 5.03) or European wild boar (8.42 ± 7.50) (Figure 2).

The mean heterozygosity per 10kb window across the autosomal genome showed that the pygmy hog has very low levels of autosomal heterozygosity (Table S3). The distribution of heterozygous peaks in pygmy hog genomes shows that 87.4% are shared by all individuals (Figure S1A). These consistent peaks of heterozygosity are strongly enriched for olfactory receptor (OR) genes (Figure S1B). It is well known that OR gene repertoires evolve rapidly through gene duplication, pseudogenization, and loss in other pig species and mammals (Paudel et al., 2013). It is likely that a large fraction of these consistent heterozygosity peaks contains inflated estimates of genetic variation caused by mismapping due to copy number variation of OR gene families. We therefore excluded regions with OR genes, which results in a lower heterozygosity distribution (Figure S1C). The 30 genes within the remaining diverse hotspots are mainly related to energy metabolism processes and immune response (Figure S1D and Table S4).

**Figure 3:**
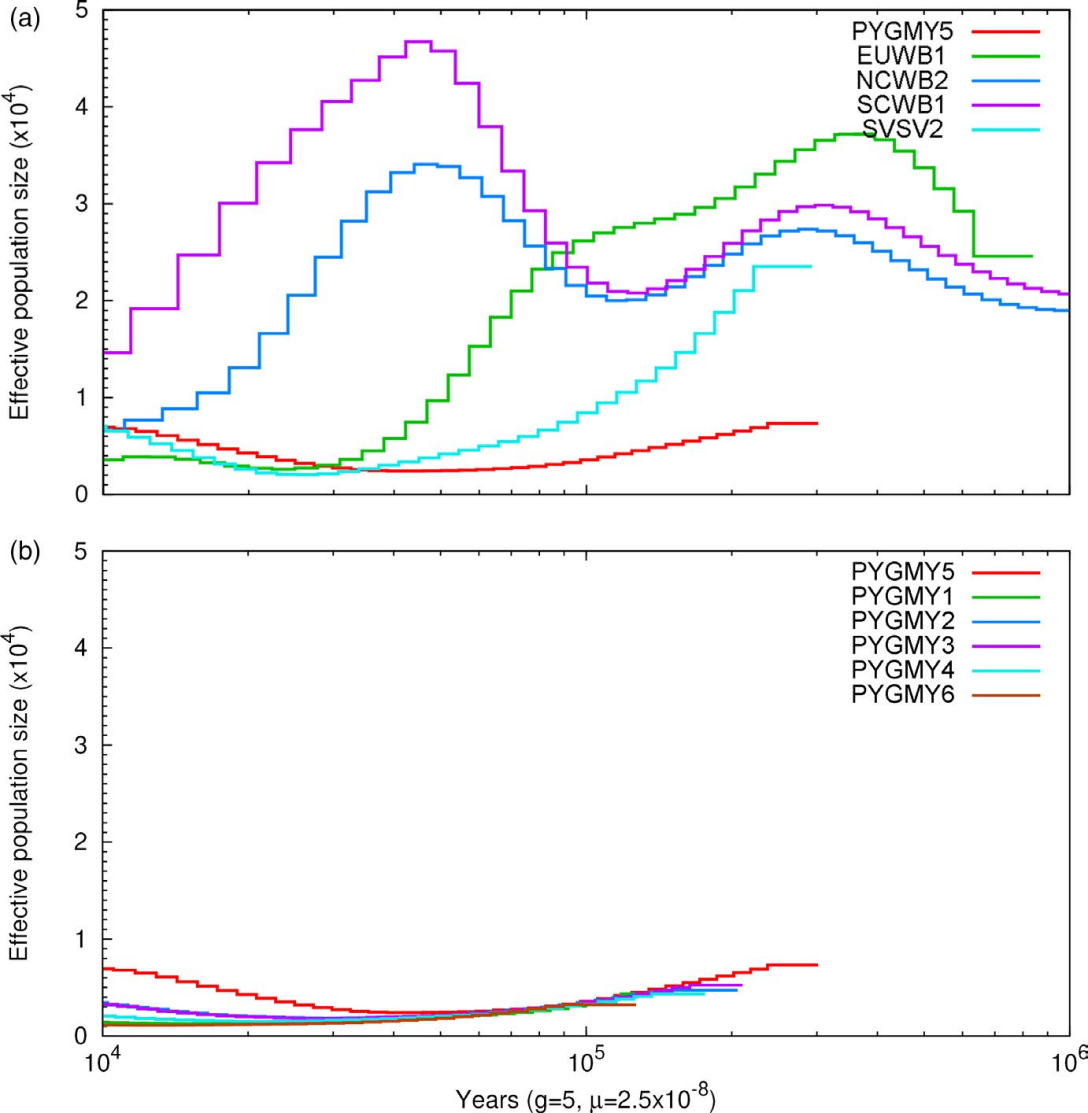
Demographic history of pygmy hogs compared to other pig species. Demographic history was inferred using a hidden Markov model (HMM) approach as implemented in pairwise sequentially Markovian coalescence (PSMC). (a). Comparison between pygmy hog and other Sus species. (b). Historical effective population size for all pygmy hog individuals. PYGMY, pygmy hog; SBSB, Sus barbatus; SVSV, Sus verrucosus; EUWB, European wild boar; NCWB, Northern China wild boar; SCWB, Southern China wild boar; for detailed sample abbreviations, see Table S1

We investigated the historical changes of effective population size within pygmy hogs and compared them with other *Suidae* species. The results of the PSMC analysis revealed a persistent low effective population size smaller than ~500 from 100,000 up to 10,000 years ago (Figure 3).

**Figure 4:**
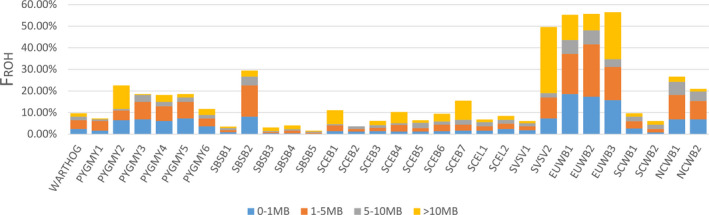
Proportion of the genome covered by ROH (F_ROH_). ROH are divided into four categories ranging from a relatively “small” (0.2–1 Mb) size category to a relatively “large” (>10 Mb) size category. WARTHOG, Phacochoerus africanus; PYGMY, pygmy hog; SBSB, Sus barbatus; SCEB, Sus cebrifons; SCEL, Sus celebensis; SVSV, Sus verrucosus; EUWB, European wild boar; NCWB, Northern China wild boar; SCWB, Southern China wild boar; for detailed sample abbreviations, see Table S1

ROH were separated into four size classes. Among the *Suidae* species, the pygmy hog has an intermediate ROH coverage (Figure 4, Figure S2). On average, we found that the captive pygmy hogs have 408 ± 190 ROH with a total coverage of 17.8 ± 4.1% (means ± SDs, equal to 422 ± 101 Mb) and that the wild pygmy hogs contain 420 ± 142 ROH with a total coverage of 23.2 ± 2.9% (576 ± 74 Mb). This average is higher for pygmy hogs than for most Island of Southeast Asia (ISEA) *Sus* species (6.3 ± 1.3%, 157 ± 32 Mb). Compared to the highly inbred *Sus verrucosus* individual (48.9%, 1,217 Mb), or European wild boars (56.0 ± 0.4%, 1,388 ± 11 Mb), the proportion of ROH in pygmy hog genomes is significantly lower. In most pygmy hog individuals, the largest proportion of the genome was covered by short ROH (size ranges of 0–1 and 1–5 Mb). Notably, one pygmy hog (PYGMY2) has significantly more long ROH than the other five individuals (*t*‐test, *p* = .04475).

### 3.3 Analysis of genetic load in pygmy HOG genomes

**Figure 5:**
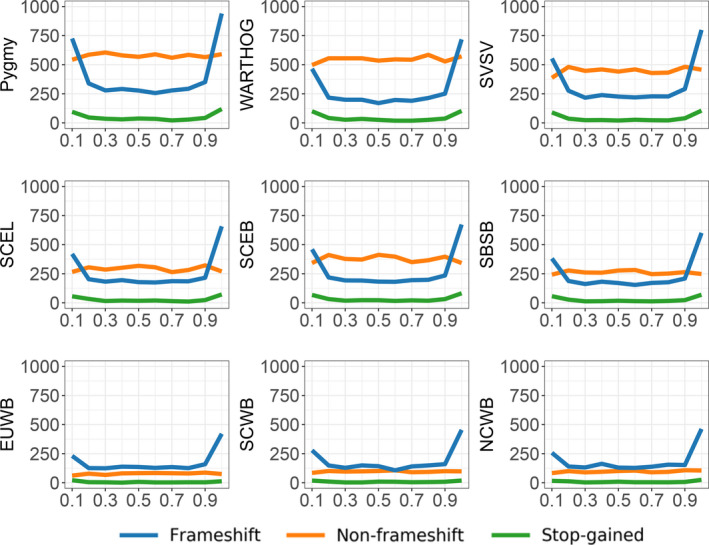
Relative position in the protein for frameshift, nonframeshift, and stop‐gained variants in the different suid population. The x‐axis displays the relative position of amino acid along the protein. The y‐axis displays the average amount of variants within populations. WARTHOG, Phacochoerus africanus; PYGMY, pygmy hog; SBSB, Sus barbatus; SCEB, Sus cebrifons; SCEL, Sus celebensis; SVSV, Sus verrucosus; EUWB, European wild boar; NCWB, Northern China wild boar; SCWB, Southern China wild boar; for detailed sample abbreviations, see Table S1

We functionally annotated the variants found in our data. Variations which are predicted to result in frameshift, stop‐gained, and deleterious missense translation were analyzed further. Compared with other species, pygmy hog harbors the highest number of frameshift, stop‐gained, and missense variants (Figure 5), and the overwhelming majority of these variants is fixed within pygmy hogs (Figure S3). The functional implication underlying the putative deleterious variants in the pygmy hog genomes was further investigated. First, to avoid uncertainty caused by alignment ambiguities, variants located in OR genes were excluded (see above). Next, to assess potential genetic load in the pygmy hog, we further extracted pygmy hog‐specific frameshift, stop‐gained, and missense mutations. Overall, 5,972 frameshift mutations, 389 stop‐gained mutations and 1,772 deleterious missense mutations were observed to be pygmy hog‐specific. To assess the potential effect of these functional variants, we used pig Combined Annotation Dependent Depletion (pCADD) scores to evaluate the predicted impact of stop‐gained and missense mutations (Groß et al., 2020). The pCADD scores are derived from a supervised classification that integrates multiple annotations, including conservation score (e.g., PhyloP, PhastCons, and GERP), and transcriptomic and epigenomic parameters (e.g., RNA‐seq and ChIP‐seq). Pygmy hogs appear to have more high‐impact mutations compared to the common warthog (*Phacochoerus africanus*), European wild boar, and Javan warty pig (Figure S4). An enrichment of frameshift, stop‐gained, and missense mutations in the N‐ and C‐terminal end of the affected genes can be observed (Figure S5A). We further predicted the distribution of high‐impact variation within protein domains. In the terminal region of proteins, we found a relatively larger proportion of variants located in protein domains (Figure S5B). Functional analysis did not reveal a significant gene ontology enrichment for frameshift and stop‐gained mutations, or for missense mutations, however, significant enrichment for genes involved in immunity and hemostasis was found (Figure S5B and Tables S5–S7).

Purging of deleterious alleles will occur naturally, as inbreeding increases the frequency of homozygotes where recessive effects are exposed to selection. However, whether the persistence of a small population size over long periods of time can be attributed to continued purging of harmful recessive mutations has not been studied. To investigate this, we conducted forward‐in‐time simulations from 100k years ago onward with different consistent effective population sizes. Our results indicated that the population size is the key factor that influences the genetic load (Figure S6). Numbers of strongly and moderately deleterious alleles were predicted to be remarkably increased in the current populations following the reduction of population size. The total number of deleterious alleles per individual in the current population relative to the ancestral population varied according to selection and dominance coefficients. Although we can still observe the elimination of harmful mutations in large populations, in small population, selection against deleterious alleles was weakened dramatically and the accumulation of additive deleterious alleles became more severe. Under an additive regime scenario, with a population size smaller than 1,000, current genomes consistently contained more deleterious alleles per individual than in the ancestral genomes. Under a recessive regime scenario, when population size is smaller than 100, we found that deleterious alleles per individual exceeded the numbers in ancestral genomes (Figure S6A,B). Moreover, all harmful mutations, including high‐impact mutations, tended to be homozygous, which is consistent with our empirical findings in the pygmy hog genomes. In sum, these findings suggest the limitation of purging of high‐impact alleles in the historically persistent small population of pygmy hogs.

## 4 DISCUSSION

This study offers insight into the historical demography and current genetic conservation status of the critically endangered pygmy hog. The continued low population size for the past one hundred thousand years, the very low genetic diversity, and the accumulation of potentially harmful mutations are supporting the endangered conservation status of this species. Being a small and isolated population, the pygmy hog has low genomic diversity and heterozygosity compared to other pig species. Although having low genetic variation similar to other critically endangered species, such as the Cheetah and the Tasmanian devil, the pygmy hog genome possesses a relative low level of ROH compared to the mentioned endangered species. In the meantime, unlike the island foxes, the effective size of pygmy hog populations has been so small for a very long time that effective purging of harmful mutations is likely impossible. This makes the pygmy hog an interesting model for studying the survival of small populations.

Demographic analyses of the pygmy hog revealed a persistent low effective population size with fewer than ~500 animals over the past one hundred thousand years to ten thousand years. These results are consistent with paleontological evidence where all fossil finds of pygmy hogs outside the Assam region were from ~1 Mya (Pickford, 2013). This suggests that the pygmy hog used to have a broader distribution range and then started contracting already during the Pleistocene. Phytogeographic analysis shows that the type of grasslands currently found at the southern foothills of the Himalayas was far more widespread across parts of South Asia during the Pliocene and early Pleistocene (Dennell, 2011; Dowsett et al., 1994). According to our PSMC analysis, the pygmy hog was not noticeably affected by the last glacial maximum (LGM), which, in contrast, had a huge effect on effective population size in the Eurasian wild boar (Groenen et al., 2012). Paleoclimatologists have hypothesized that the southern flank of the Himalayas during the LGM harbored a range of climatological refuges (Singh et al., 2010). This would continue to provide a suitable habitat, allowing pygmy hog to continue to have a constant, local, population size.

After persisting a long period of low effective population size, the current pygmy hog population is harboring more deleterious mutations, or precisely high‐impact mutations, than other *Suidae* species. Notably, reference bias can influence variant calling by missing alternative alleles or by wrongly calling heterozygous sites as homozygous for the reference allele (Ros‐Freixedes et al., 2018). This effect increases with the genetic distance toward the reference genome (*Sus scrofa*) (Liu et al., 2019). Although we do expect some bias in this estimation of high‐impact mutations in pygmy hogs, caused by the genetic distance to the reference genome, the pygmy hog does harbor more high‐impact mutations than the warthog (Figure 5; Figure S4). Since the African warthog is even more distantly related to *Sus scrofa*, distance to the reference genome alone cannot explain the high frequency of high‐impact mutations in the pygmy hog. Therefore, the pygmy hog appears to have a dramatically increased rate of accumulation of high‐impact mutations.

In pygmy hog genomes, high‐impact mutations show a pattern of historical purifying selection, since most of them are located at the N‐ and C‐terminal end of genes. However, even within the two tails of proteins, which generally contain less functional domains, there are still abundant mutations that may influence the function of the protein. The gene set enrichment analysis clearly shows that certain GO terms are strongly associated with pygmy hog‐specific missense mutations. These GO terms are mostly related to the immune response and blood coagulation pathways. In the meantime, selection against deleterious recessive alleles is less efficient when population size is small (García‐Dorado, 2012). A previous study suggested that the minimum effective population size to avoid severe inbreeding depression in the short term is Ne ≈ 70 (Caballero et al., 2017). This is consistent with our simulations, which show an elevation of deleterious mutations in small populations. Moreover, the majority of the deleterious mutations is in the homozygous state, suggesting that these are fixed and the accumulation of deleterious mutations is exceeding the purging effect. The overall ROH coverage in pygmy hog indicated a low level of recent inbreeding. Such dynamic relationship between inbreeding and purging has thus far not been observed in other endangered populations illuminating the importance of species‐specific genetic analysis for predicting and enhancing population persistence. The results corroborate the assumption that many pygmy hog‐specific variants are predominantly harmful, or greatly affect gene functioning. High‐impact mutations can also show selective advantage by genetic hitchhiking in regions under selection, sometimes even boosting the fitness in specific lineage due to the local adaptation (Bosse, 2019; Bosse et al., 2019). With the limited information we have, it is difficult to assess the actual effect of specific alleles, some of which potentially could be related to shaping species characteristic like, for example, behavioral traits and adaptation to a specific habitat. However, since the accumulation of deleterious mutations could exceed purging in such demographic scenario, we believe that the majority of these predicted high‐impact variants have a negative effect on fitness.

The current pygmy hog population exhibits low nucleotide diversity and heterozygosity compared to other pig species, which conforms to its critically endangered status. Comparing the mitochondrial genomes of three wild‐caught pygmy hogs and three captive individuals, we find that there is no variation within the analyzed samples. The small sampling size, six individuals in this case, may lead to sampling biases of maternal linage. The wild individuals and founders of the captive population were independently sampled in 2014 and 1996, respectively, reducing the possibility of sampling biases to a certain extent. This indicates a very low mitochondrial DNA diversity and a potential maternal bottleneck before the establishment of the captive population. Severe unbalanced sex ratio is often observed in species on the edge of extinction (Allentoft et al., 2010; Bessa‐Gomes et al., 2004; Pečnerová et al., 2017). The same situation may have happened to pygmy hogs in the 60s, when they almost disappeared from the wild.

The long‐term small population size and potential historical bottleneck lead to reduction in genetic diversity, which further limits the ability to adapt to environmental changes. By comparison, the close relative to the pygmy hog, *Sus scrofa*, is widely distributed over Eurasia continent, whereas the pygmy hog is highly specialized, only living in the tall grass savannah. A conservation program was used to transfer pygmy hogs to the Zurich and London Zoos in 1998 and 1876, respectively (Oliver & Roy, 1993), but both failed. The dependence on a specific ecological niche and a reduced adaptability to environmental changes could be the consequence of the reduced standing genetic variation and accumulation of genetic load. Thus, maximum efforts should be made to protect the fragile high‐grassland ecosystem.

While genome‐wide allelic diversity may be low, the pygmy hog does not show extreme ROH coverage. Specifically, long ROH are rare, compared to, for instance, the sequenced male Javan warty pig (SVSV01M01) or European wild boars, which are known to have gone through series of recent population bottlenecks (Bosse et al., 2012; Groenen et al., 2012). Between wild and captive pygmy hog populations, there is no significant difference in the total length of ROH. The overall ROH landscape in pygmy hog indicates very little recent inbreeding. The observed ROH were possibly caused by an ancient bottleneck followed by a gradual breakdown of ancient long ROH (Speed & Balding, 2015). Notably, although the pedigree information does not indicate closely related mating, one of the pygmy hogs (PYGMY2) has significantly more long ROH than the other individuals. Thus, the founders of the maternal and the paternal lineage of PYGMY2 seem to share more relatedness than the founders of other two captive individuals. This result is a warning that the assumption in conservation practices that wild captured founders are genetically unrelated is not always valid.

Considering the genetic diversity and inbreeding level, the initial founders of the PHCP were sufficiently representatives of the wild population. However, the captive individuals in this study represent the third and fourth generation of the breeding program. The observation that the most recent generation showed a significant decrease in individual heterozygosity indicates that drift effects likely are becoming prominent after more than five generations (Purohit et al., 2019). Since there is no other existing wild population and pygmy hog is the only member in its genus, “genetic rescue” is not feasible for the pygmy hog population. Fortunately, signatures of very recent inbreeding, compared to some of the other endangered pig populations, are relatively mild. Furthermore, no noticeable morphological changes have been reported (i.e., length, weight, external appearance) (Deka et al., 2009; Narayan et al., 1999). Other additional phenotypic traits have not yet been examined within this population, and therefore, it is not known whether the low levels of genetic diversity are impacting population fitness. Considering the recent decline of genetic diversity in captive pygmy hogs (Purohit et al., 2019), genetic defects may become apparent due to the recessive deleterious alleles being homozygous. Recent studies have shown the potential of using genomics information to monitor deleterious mutations in breeding program (Charlier et al., 2016; Derks et al., 2018, 2019). A genomic method to measure the kinship in captivity is pressingly needed for the PHCP to prevent close relatives from mating and to estimate individual genetic load to guide the artificial selection against harmful mutations causing genetic defects. To preserve the evolutionary potential of the pygmy hog population, it is essential to enlarge the extant population and prevent further decline of population size due to disease outbreaks or anthropogenic threats.

## 5 CONCLUSION

In conclusion, long‐term persistence of extremely small population size can lead to an increase in genetic load. Although species management through breeding programs can prevent the occurrence and expression of harmful alleles, genetic diversity cannot be boosted by human intervention, but only by natural mutation and introgression with closely related species. Monitoring the individual heterozygosity of subsequent generations is, hence, crucial for maintaining the genetic diversity in captive pygmy hogs and to inform future conservation breeding decisions.

## DATA AND SOFTWARE AVAILABILITY

The authors declare that all data and software supporting the findings of this study are available within the article and its Supplementary Information files, or from the corresponding author upon request. Raw reads of all samples used in this study have been deposited in the European Nucleotide Archive (ENA) under accession numbers ERP001813, ERP112560, and ERP118195.

## AUTHOR CONTRIBUTIONS

M.A.M.G., O.M., M.B., and L.L. designed the study. L.L. analyzed the data. M.D.V. preformed the preliminary analyses. L.L. wrote the manuscript. O.M., M.B., H.‐J.M., M.A.M.G., and M.D.V. provided valuable suggestion and comments to improve the manuscript.

## Supporting information

Fig S1‐S6

Table S1‐S7
