# Supplementary material for: Genetic consequences of long‐term small effective population size in the critically endangered pygmy hog
Source: Evol Appl. 2020 Nov 11;14(3):710–20. doi: 10.1111/eva.13150 (PMC7980308; doi:10.1111/eva.13150)
Supplement: Supplementary file 1 — Fig S1‐S6 [file EVA-14-710-s001.docx]

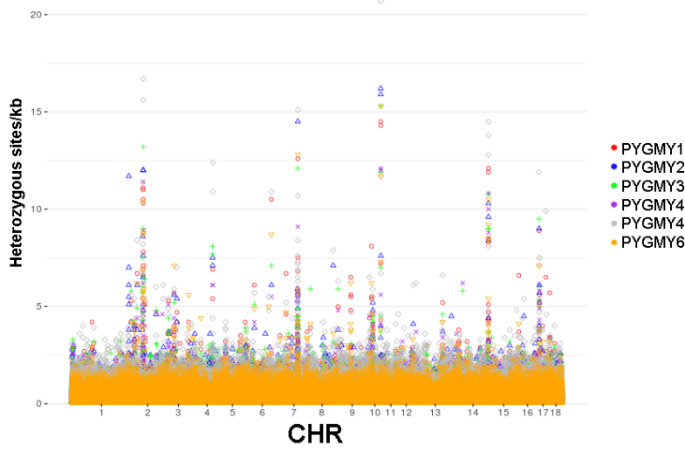

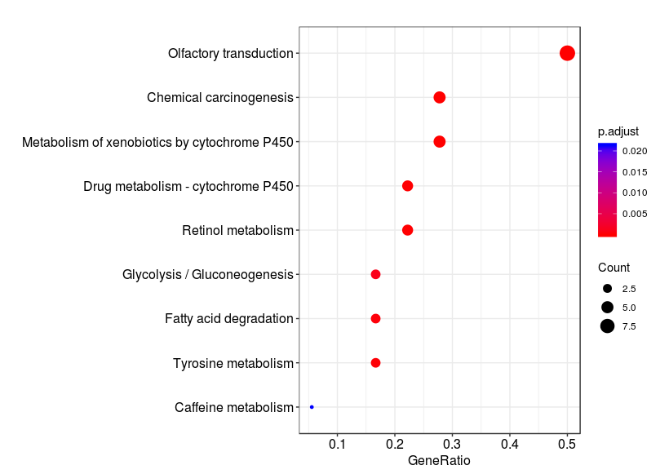


a

b


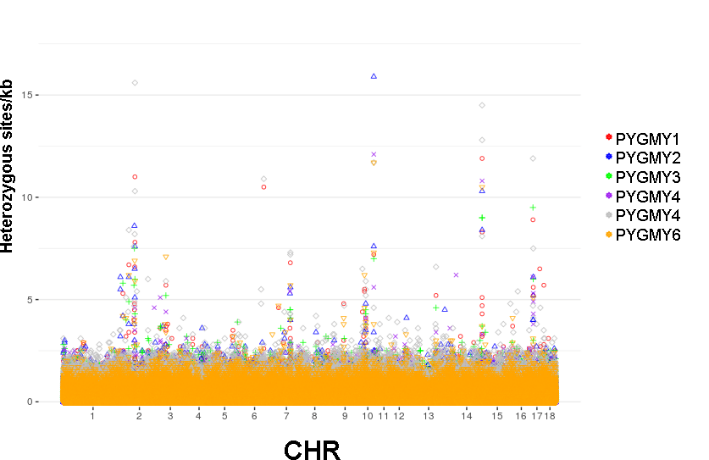

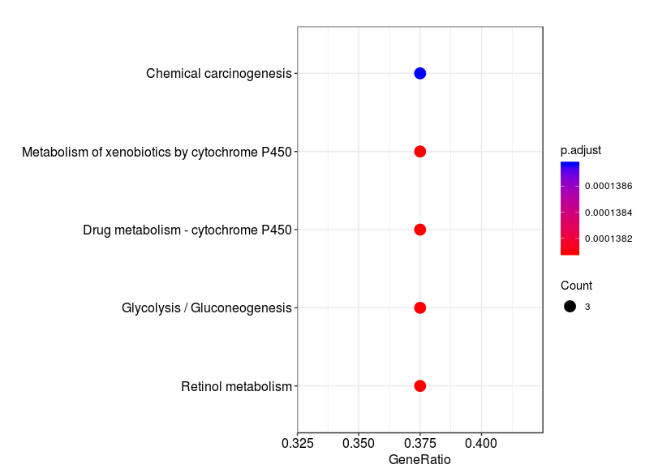


d

c

Sup Fig. 1 a) Heterozygosity distribution of pygmy hog. Heterozygosity in 100Kb windows with a 10Kb step across the autosomal genome shows the genomic flatlining punctuated by occasional peaks of heterozygosity in pygmy hogs. Sample abbreviations see Sup table 1. b) KEGG functional enrichment result for gene set located within pygmy hog heterozygous hotspot. The number of horizontal axis is the number of enriched genes. The bubble size indicates the ratio of genes in each term or pathway, and different colours correspond to different adjusted p-values. The p-values are adjusted by Benjamini-Hochberg method. c) Heterozygosity distribution of pygmy hog, excluding windows with OR genes. Heterozygosity in 100Kb windows with a 10Kb step across the autosomal genome shows the genomic flatlining punctuated by occasional peaks of heterozygosity in pygmy hogs. Sample abbreviations see Sup table 1. KEGG functional enrichment result for gene set located within pygmy hog heterozygous hotspot, excluding windows with OR genes. d) The number of horizontal axis is the number of enriched genes. The bubble size indicates the ratio of genes in each term or pathway, and different colours correspond to different adjusted p-values. The p-values are adjusted by Benjamini-Hochberg method.


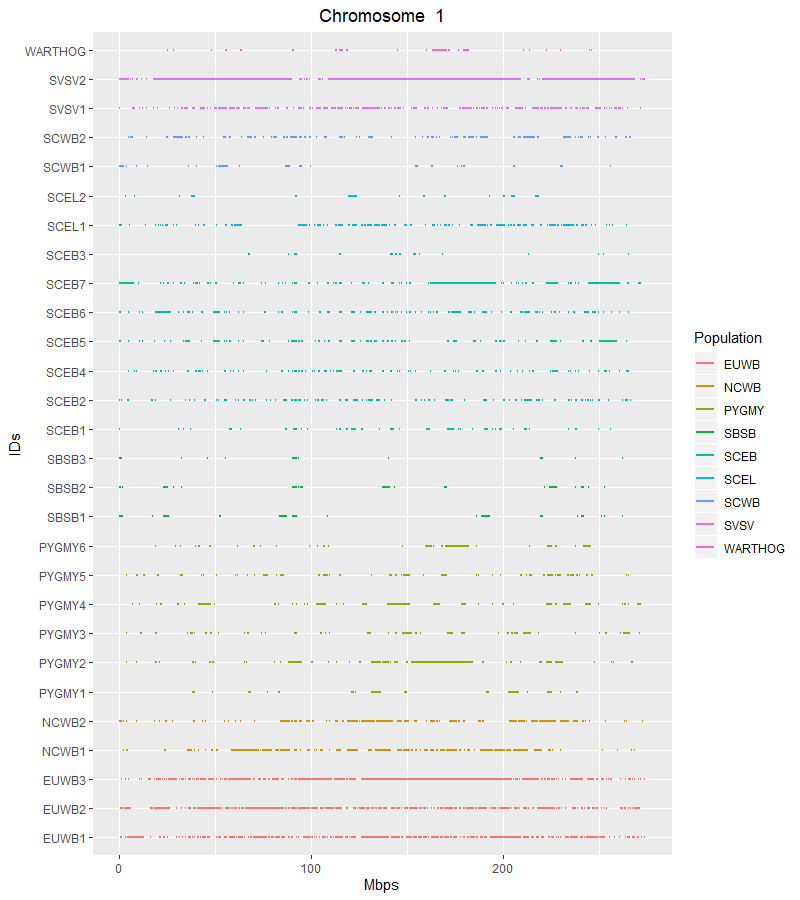


Sup Fig. 2 Physical distribution of ROH over chromosome 1.


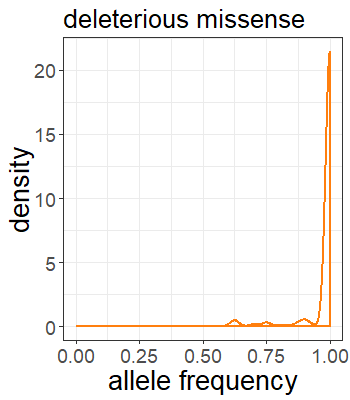

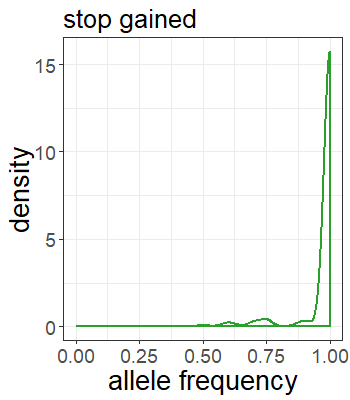

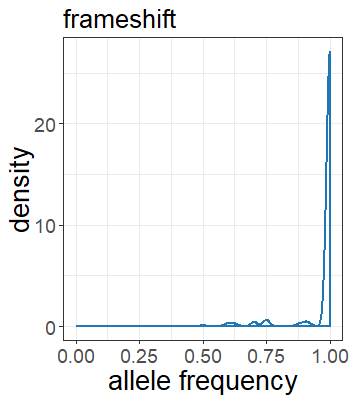


Sup Fig. 3 Allele frequency distribution for frameshift, deleterious missense, and stop-gained variants.


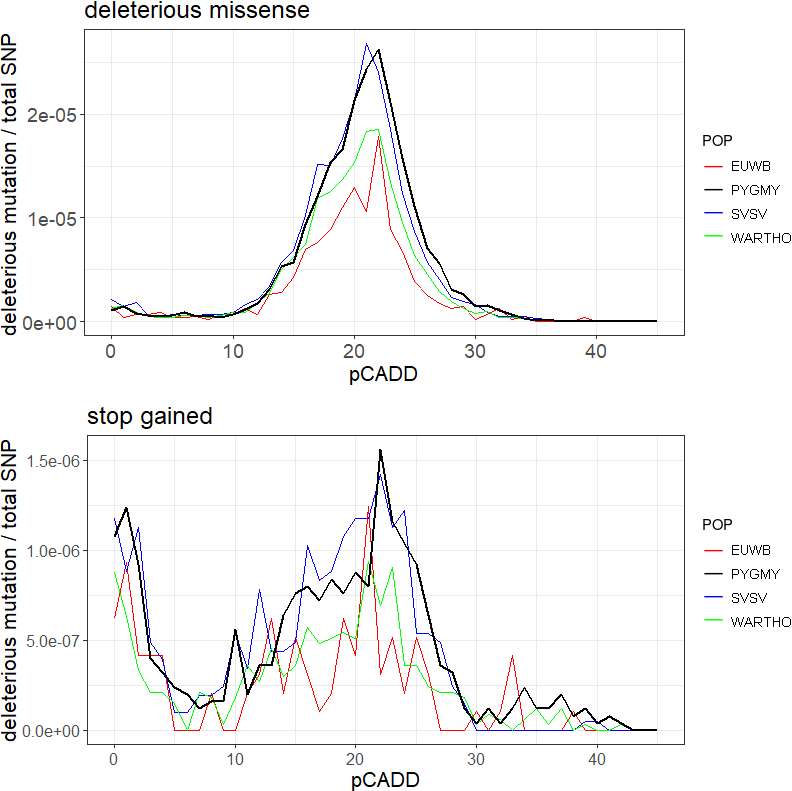


Sup Fig. 4 Distribution of pig CADD score (pCADD) of deleterious missense and stop gained mutation.

a

b


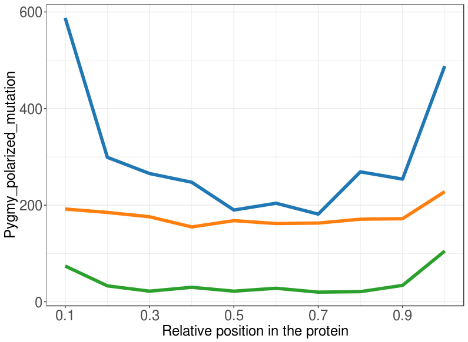

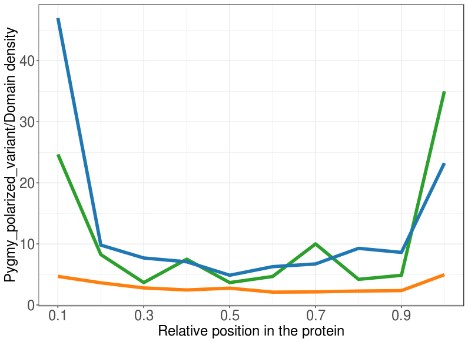


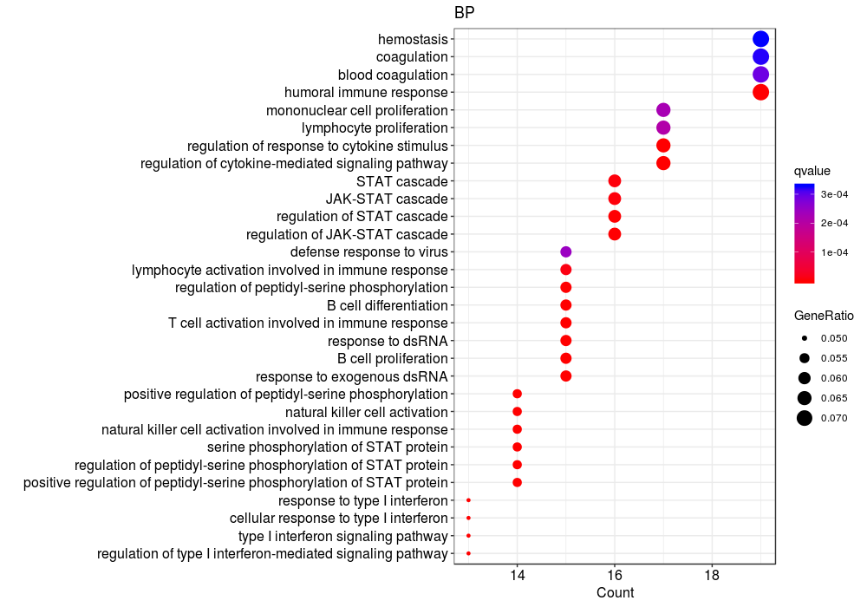


c

Sup Fig. 5 a) Relative position in the protein for pygmy hog specific frameshift, non-frameshift, and stop-gained variants. The x-axis displays the relative position of amino acid along the protein. b) Distribution for pygmy hog specific frameshift, non-frameshift, and stop-gained variants related to protein domain. The y-axis displays counts of variant located in a protein functional domain. The x-axis displays the relative position of amino acid along the protein normalized by the distribution of protein domain. Information of functional domain was downloaded from <https://pfam.xfam.org/>. c) Functional enrichment result for gene set harbored missense mutations. The number of horizontal axis is the number of enriched genes. The bubble size indicates the ratio of genes in each term or pathway, and different colours correspond to different adjusted p-values. The p-values are adjusted by Benjamini-Hochberg method.


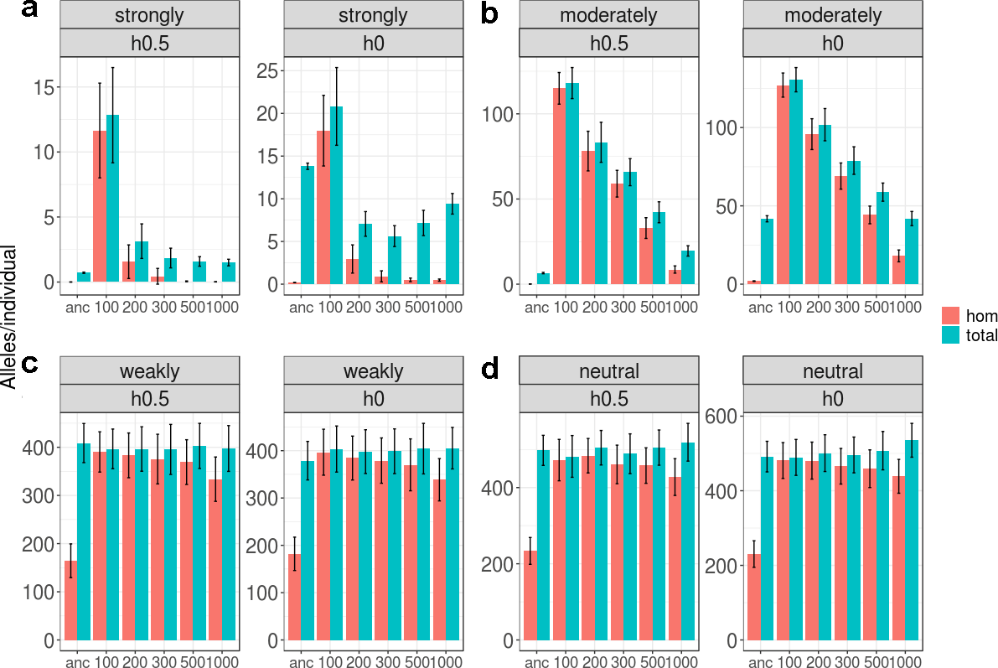


Sup Fig. 6 Forward simulations of genetic variation in pygmy hog population. Charts shows the total number of derived alleles and the number of homozygous derived alleles per individual (Y-axis) by dominance and selection strength. Bar height represents the mean across all 50 replicates. Selection strengths are set as four categories: a) strongly deleterious, b) moderately deleterious, c) weakly deleterious, and d) neutral. Simulations include a mixture of neutral and deleterious alleles in which deleterious alleles are either entirely additive (additive regime, h0.5) or entirely recessive (recessive regime, h0). Anc is the ancestral population and the numbers (100-1000) represent the population size used in the simulations.
